# Global Prevalence of *Anaplasma phagocytophilum* in Cattle: A One Health Perspective, Meta‐Analysis and Future Predictions (up to 2035)

**DOI:** 10.1002/vms3.70251

**Published:** 2025-02-19

**Authors:** Amir Abdoli, Meysam Olfatifar, Leila Zaki, Farhad Nikkhahi, Fatemeh Fardsanei, Sona Sobhani, Hamid Sadeghi, Aida Vafae Eslahi, Milad Badri

**Affiliations:** ^1^ Zoonoses Research Center Jahrom University of Medical Sciences Jahrom Iran; ^2^ Department of Parasitology and Mycology Jahrom University of Medical Sciences Jahrom Iran; ^3^ Gastroenterology and Hepatology Diseases Research Center Qom University of Medical Sciences Qom Iran; ^4^ Medical Microbiology Research Center Qazvin University of Medical Sciences Qazvin Iran

**Keywords:** *Anaplasma phagocytophilum*, global prevalence, meta‐analysis, tick‐borne diseases

## Abstract

*Anaplasma phagocytophilum* is an emerging tick‐borne zoonotic bacterium, which is considered a significant risk to the health and industry of cattle in tropical and sub‐tropical regions worldwide. This research focuses on examining the worldwide occurrence of *A. phagocytophilum* in cattle. Several databases, including Scopus, PubMed, ProQuest, Web of Science, ScienceDirect, and Google Scholar, were searched for publications spanning October 2004 to November 2024. The pooled prevalence was calculated with a 95% confidence interval (CI) using a random‐effects model based on the Freeman‐Tukey double arcsine transformation. A total of 72 studies satisfied the inclusion criteria, revealing a global prevalence of *A. phagocytophilum* in cattle estimated at 8.5% (5.9%–11.5%). Mongolia (51.9%, 45.9%–56.2%) and Guatemala (51%, 41.2%–60.7%) were countries that accounted for the highest prevalence. Moreover, the infection was most prevalent in African region with prevalence of 11.3% (3.9%–21.5%). The highest prevalence rate was observed in hot‐summer Mediterranean climate (13.7%, 4.7%–26.2%). The analysis indicated that immunological techniques were associated with the highest prevalence rate (14.2%, 6.5%–24.3%). The findings of the present research highlighted important geographical and environmental factors that affect the prevalence of disease. In the fields of veterinary medicine and public health, these findings enhance disease management plans and preventative initiatives.

## Graphical abstract

GRAPHICAL ABSTRACT

✓ Bovine anaplasmosis is a major tick‐borne disease affecting cattle herds.

✓ The global prevalence of *Anaplasma phagocytophilum* in cattle was 8.5%.

✓ Mongolia and Guatemala were countries accounted for the highest prevalence.

✓ *Anaplasma phagocytophilum* was most prevalent in cattle African region and Hot‐summer Mediterranean climate.

✓ Understanding infection dynamics in cattle is key to effective control, protecting animal health and sustaining agricultural productivity.

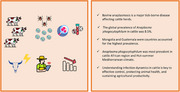

## 1 Background

Bovine anaplasmosis is a major tick‐borne illness that impacts cattle populations, resulting from infections by different species of *Anaplasma*, such as *A. marginale*, *A. bovis*, *A. centrale* and *A. phagocytophilum* (Altay et al. 2022). It is transmitted mainly by ixodid tick bites or mechanical transfer of newly infected red blood cells. The latter can occur by biting flies or using various surgical instruments such as needles, or equipment utilized in procedures such as dehorning, castration or tattooing (de la Fuente et al. 2005; Rizzoli et al. 2014; Aubry and Geale 2011). *A. phagocytophilum* is a zoonotic gram‐negative bacterium that obligatorily inhabits the cytoplasm of host cells, and it belongs to the family Anaplasmataceae (previously known as *Ehrlichia equi* and *Ehrlichia phagocytophila*) (Dumler et al. 2001; Woldehiwet 2010).

So far, *A. phagocytophilum* is documented in humans, ruminants like cattle (*Bos taurus*), sheep (*Ovis aries*), goats (*Capra hircus*), horses (*Equus caballus*) and deer species such as red deer (*Cervus elaphus*), roe deer (*Capreolus capreolus*), white‐tailed deer (*Odocoileus virginianus*) and moose (*Alces alces*). It has also been reported in carnivores, including dogs (*Canis lupus familiaris*), cats (*Felis catus*) and red foxes (*Vulpes vulpes*), as well as other species such as wild boar (*Sus scrofa*), hedgehogs (*Erinaceus europaeus*), rodents and birds (Apaa et al. 2023; Bianchessi et al. 2023). It is the pathogen responsible for causing tick‐borne fever (TBF) in ruminants and granulocytic anaplasmosis in humans (HGA), horses (EGA), dogs and cats (Dugat et al. 2017; Woldehiwet 2006; Stuen, Granquist, and Silaghi 2013).

*Ixodes ricinus* is the primary carrier of *A. phagocytophilum* (Beugnet and Marié 2009; Blanco and Oteo 2002). However, *A. phagocytophilum* DNA has also been identified in other tick species, such as *Dermacentor marginatus* (Bonnet et al. 2013), *Dermacentor reticulatus* (Tomanović et al. 2013), *Haemaphysalis punctata* (Palomar et al. 2015), *Haemaphysalis concinna* (Tomanović et al. 2013) and other *Ixodes* species, including *I. persulcatus*, *I. trianguliceps* (Rar et al. 2014), *I. canisuga* and *I. hexagonus* (Keyte et al. 2021).

Anaplasmosis is prevalent in several regions worldwide, consistently impacting the dairy production sector (Paramanandham et al. 2019).

*A. phagocytophilum* exhibits a widespread distribution, with endemic regions including some specific areas in the United States, Europe and Asia (such as China, Siberian Russia and Korea), where the presence of *Ixodes persulcatus* ticks aligns with the occurrence of this bacterium (El Hamiani Khatat et al. 2021).

It was first reported and described as a febrile illness in North America in 1994 and was named human granulocytic ehrlichiosis (Bakken et al. 1994).

The primary factor contributing to the occurrence to bovine anaplasmosis and TBF is transporting of vulnerable domestic animals from tick‐free regions to areas where the disease is prevalent. In the case of HGA, engaging in recreational activities with a high risk of tick exposure or other habits that increase susceptibility, along with blood transfusion, are significant risk factors. Once infected, individuals can carry the infection for their entire life. Clinical anaplasmosis diagnosis typically involves examining stained blood smears (Atif 2015).

Microscopic techniques have proven effective in identifying animals with clinical infections. However, these methods are not efficient for identifying reservoir animals (Aubry and Geale 2011; Kocan et al. 2010). Enzyme‐linked immunosorbent assay (ELISA), indirect fluorescent antibody test (IFAT) and complement fixation test (CFT) are frequently employed in the diagnosis of bovine anaplasmosis in extensive epidemiological studies and eradication initiatives. Despite their widespread use, these techniques are unreliable for detecting of the causative agents of bovine anaplasmosis (Altay et al. 2022). Molecular identification tools such as polymerase chain reaction (PCR), reverse line blot analysis (RLB), PCR‐restriction fragment length polymorphism (RFLP) and DNA sequencing have become preferred methods for the detecting of bovine anaplasmosis, primarily owing to their heightened specificity. Furthermore, these molecular techniques can identify previously unrecognized *Anaplasma* genotypes or strains (Altay et al. 2022; Bezerra‐Santos et al. 2021).

The application of molecular tools, specifically those targeting various genes such as groEL and 16S rRNA, has facilitated the identification of multiple variants of *A. phagocytophilum* (Silaghi et al. 2011; Huhn et al. 2014).

Two novel variants associated with *A. phagocytophilum* have been identified in sheep, goats, cattle, deer, small mammals such as *Rattus rattus*, and various tick species in Italy, Turkey, Japan, China and Tunisia through the use of PCR, PCR‐RFLP and DNA sequencing (Altay et al. 2022).

Tetracyclines, including oxytetracycline, enrofloxacin and doxycycline, are essential for treating animal and human anaplasmosis (Atif, Hussain, and Mehnaz 2021).

Bovine anaplasmosis is a temporary, mild infection associated with reduced appetite, weakness and secondary infections attributable to immunosuppression (Woldehiwet 2006). High morbidity linked to abortion and a decline in milk production, leading to economic losses, has also been documented in dairy cattle with bovine anaplasmosis (McFadzean et al. 2021; Van Loo et al. 2023). Nevertheless, the precise involvement of *A. phagocytophilum* in the development and identification of abortions and stillbirths in cattle remains unclear (Van Loo et al. 2023). Bovine anaplasmosis caused by *A. phagocytophilum* is recognized as a severe clinical ailment in cattle globally. Factors contributing to the disease, such as the time of year, presence of tick vectors, geographical latitude and farm hygiene practices, have been identified as associated risk factors (Noaman 2020).

Our primary objective in conducting this comprehensive review and meta‐analysis is to evaluate the worldwide prevalence of *A. phagocytophilum* in cattle. Through meticulous analysis and synthesis of existing research, we aim to offer valuable insights into the distribution of this pathogen and identify associated risk factors that contribute to its prevalence. By systematically reviewing available literature and conducting a meta‐analysis, we strive to enhance our understanding of the epidemiology of *A. phagocytophilum* in cattle, ultimately contributing to developing informed disease management and prevention strategies. This endeavour seeks to consolidate current knowledge and aims to fill gaps in understanding, fostering advancements in the field of veterinary research and public health.

## 2 Materials and Methods

### 2.1 Approach to the Search

The current study was conducted following the guidelines outlined in the Preferred Reporting Items for Systematic Reviews and Meta‐Analyses (PRISMA) checklist (Page et al. 2021). Several databases, including Scopus, PubMed, ProQuest, Web of Science, ScienceDirect, and Google Scholar, were searched. Additionally, a manual search was conducted for articles published between October 2004 and November 2024. Search terms using AND and/or OR Boolean operators were as follows: *Anaplasma* spp., *A. phagocytophilum*, *A. phagocytophilum*, anaplasmosis, *E phagocytophila*, ehrlichiosis, tick‐borne bacterial diseases, tick‐borne pathogens, cattle, prevalence, frequency, global and worldwide.

The reference lists of the obtained publications were examined for additional research that could not be found through database searches, in addition to eliminating duplicates and unnecessary studies. Two authors independently handled the evaluation of full‐text articles and the screening of each article's abstract and title.

### 2.2 Screening and Eligibility of the Study

To sort and remove duplicates, all of the retrieved articles were mostly imported into the EndNote citation manager program (version 8, Thomson Reuters, Stamford, CT, USA). First author, year of publication, countries, WHO regions, sampling time, sample size, number of positive samples, Human Development Index (HDI), climate, humidity, annual precipitation, average temperature, annual rainfall and diagnostic technique were all gathered from retrieved articles using Microsoft Excel version 2016 (Tables 1 and 2).

**TABLE 1:** Main characteristics of the included studies reporting the prevalence of *Anaplasma phagocytophilum* in cattle.

Study no.	Author name	Publication years	Country	Continent	Time of sampling	Sample size	Positive	Season	Type of ticks	References
1	Hulínská et al.	2004	Czech Republic	Europe	2002–2003	110	6	—	*Ixodes ricinus*	Hulinská et al. (2004)
2	Hofmann‐Lehmann	2004	Switzerland	Europe	2002	285	2	Summer	*Ixodes ricinus*	Hofmann‐Lehmann et al. (2004)
3	Stuen et al.	2005	Norway	Europe	2004	58	20	Spring and Summer	—	Stuen et al. (2005)
4	Chahan et al.	2005	China	Asia	2004	146	15	—	—	Chahan et al. (2005)
5	De La Fuente et al.	2005	Italy	Europe	—	50	13	—	*Rhipicephalus bursa* *Rhipicephalus turanicus* *Haemaphysalis punctata* *Hyalomma m. marginatum* *Dermacentor marginatus* *Rhipicephalus sanguineus* *Hyalomma m. lusitanicum* *Ixodes ricinus*	de la Fuente et al. (2005)
6	Teglas et al.	2005	Guatemala	North America	2003	96	49	Summer	*Rhipicephalus microplus* *Amblyomma cajennense*	Teglas et al. (2005)
7	Amusategui et al.	2006	Spain	Europe	—	456	14	—	—	Amusategui, Sainz, and Tesouro (2006)
8	Torina et al.	2007	Italy	Europe	2003–2005	246	40	—	—	Torina and Caracappa (2007)
9	Gokce et al.	2008	Turkey	Asia	2002	1440	183	Spring and Autumn	—	Gokce et al. (2008)
10	Ebani et al.	2008	Italy	Europe	2004–2007	137	23	Winter, Spring and Summer	—	Ebani et al. (2008)
11	Ooshiro et al.	2008	Japan	Asia	2006	15	6	—	—	Ooshiro et al. (2008)
12	Jilintai et al.	2009	Japan	Asia	2007	78	1	Autumn	—	Jilintai et al. (2009)
13	Chae et al.	2009	Korea	Asia	2002–2004	129	1	—	—	Chae et al. (2009)
14	Noaman and Shayan	2009	Iran	Asia	2007	150	2	Spring and Summer	—	V. Noaman and Shayan (2009)
15	Muhanguzi et al.	2010	Uganda	Africa	2008	375	10	Winter and Spring	—	Muhanguzi et al. (2010)
16	Aktas et al.	2011	Turkey	Asia	2008	389	3	Summer and Autumn	—	Aktas, Altay, and Dumanli (2011)
17	Murase et al.	2011	Japan	Asia	—	1251	42	—	*Ixodes persulcatus*	Murase et al. (2011)
18	Guyot et al.	2011	Belgium	Europe	2005	30	2	Spring	—	Guyot et al. (2011)
19	Zhang et al.	2012	China	Asia	2007–2010	433	3	—	*Haemaphysalis longicornis* *Dermacentor silvarum* *Haemaphysalis concinna* *Ixodes persulcatus*	L. Zhang et al. (2012)
20	Ayling et al.	2012	England	Europe	2011	8	1	Spring	—	Ayling et al. (2012)
21	Ybañez et al.	2013	Japan	Asia	2011	50	1	Spring	*Ixodes persulcatus*	Ybañez et al. (2013)
22	Yang et al.	2013	China	Asia	2010	20	7	Summer	*Haemaphysalis qinghaiensis*	Yang et al. (2013)
23	Ceci et al.	2014	Italy	Europe	2000	1500	15	Spring, Summer and Autumn	*Rhipicephalus bursa* *Ixodes ricinus, Boophilus annulatus* *Hyalomma marginatum, Dermacentor marginatus, Haemaphysalis sulcata* *Haemaphysalis parva* *Haemaphysalis inermis* *Haemaphysalis punctata*	Ceci et al. (2014)
24	Belal et al.	2015	Bangladesh	Asia	2013–2014	395	102	Winter, Spring, Summer and Autumn	—	Belal, Al Mahmud, and Ferdous (2014)
25	Aktas et al.	2015	Turkey	Asia	2014	133	24	—	—	Aktas and Özübek (2015)
26	Dahmani et al.	2015	Algeria	Africa	2013	36	15	—	—	Dahmani et al. (2015)
27	Hoşgor et al.	2015	Turkey	Asia	2006–2007	679	186	Winter, Spring, Summer and Autumn	*Hyalomma marginatum* *Hyalomma excavatum*	Hosgör et al. (2015)
28	Yang et al.	2015	China	Asia	2012	125	8	Summer	—	Yang et al. (2015)
29	Cho et al.	2016	Korea	Asia	—	119	1	—	—	Cho et al. (2016)
30	Noaman et al.	2016	Iran	Asia	—	209	2	—	—	V. Noaman et al. (2016)
31	Andersson et al.	2017	Sweden	Europe	—	71	10	—	—	Andersson et al. (2017)
32	Dugat et al.	2017	France	Europe	2013–2015	998	6	—	*Dermacentor marginatus* *Hyalomma marginatum* *Rhipicephalus* spp.	Dugat et al. (2017)
33	Hussain et al.	2017	Pakistan	Asia	—	148	9	—	—	Hussain et al. (2017)
34	Said et al.	2017	Tunisia	Africa	2015	367	7	—	—	Ben Said et al. (2017)
35	Seo et al.	2018	Korea	Asia	2016	764	20	—	—	Seo et al. (2018)
36	Von Fricken et al.	2018	Mongolia	Asia	2014–2015	364	186	Winter, Spring, Summer and Autumn	—	von Fricken et al. (2018)
37	Han et al.	2018	Korea	Asia	2016–2017	214	2	Spring, Summer and Autumn	—	Han et al. (2018)
38	Teshale et al.	2018	Ethiopia	Africa	—	457	19	—	—	Teshale et al. (2018)
39	Vasić et al.	2018	Serbia	Europe	2013	163	4	Spring and Summer	*Dermacentor marginatus, Haemaphysalis punctata, Ixodes ricinus*	Vasić et al. (2018)
40	Silaghi et al.	2018	Germany	Europe	2011–2012	58	16	Winter, Spring, Summer and Autumn	—	Silaghi et al. (2018)
41	Abd El‐Baky and Allam	2018	Egypt	Africa	2014–2016	40	6	—	*Amblyomma gemma* *Amblyomma lepidum, Amblyomma variegatum, Rhipicephalus annulatus* *Hyalomma albiparmatum* *Hyalomma excavatum* *Hyalomma dromedarii* *Hyalomma impeltatum* *Rhipicephalus pulchellus, Rhipicephalus sanguineus*	Allam, El Moghazy, and Abdel‐Baky (2018)
42	Salehi‐Guilandeh et al.	2019	Iran	Asia	2015	150	3	Spring, Summer and Autumn	—	Salehi‐Guilandeh et al. (2019)
43	de Jesus Fernandes et al.	2019	Mozambique	Africa	2014	219	6	Summer	—	de Jesus Fernandes et al. (2019)
44	Iqbal et al.	2019	Pakistan	Asia	2018	450	12	Summer	—	Iqbal et al. (2019)
45	Mabizari	2019	Puerto Rico	North America		198	1	—	—	Mabizari (2019)
46	Zhou et al.	2019	China	Asia	2016–2017	345	17	Winter, Spring, Summer and Autumn	—	Z. Zhou et al. (2019)
47	Ayyez et al.	2019	Iraq	Asia	2014–2016	400	50	—	—	Ayyez, Khudhair, and Kshash (2019)
48	Yan et al.	2020	China	Asia	2015	493	12	Winter, Spring and Summer	—	Yan et al. (2020)
49	Ajel and Mahmood	2020	Iraq	Asia	2020	200	15	Winter, Spring, Summer and Autumn	—	Ajel and Mahmood (2020)
50	Noaman	2020	Iran	Asia	2017–2018	1851	286	Winter, Spring, Summer and Autumn	—	V. Noaman (2020)
51	Nouri et al.	2020	Iran	Asia	2018	200	66	Winter, Spring, Summer and Autumn	—	N. Vahedi Nouri, Noaman, and Abadi (2020)
52	Vahedi Nouri and Noaman	2021	Iran	Asia	2016	105	21	Autumn	—	N. Vahedi Nouri and Noaman (2021)
53	Barradas et al.	2021	Angola	Africa	2019	98	2	Winter	*Amblyomma variegatum, Rhipicephalus decoloratus* *Rhipicephalus evertsi mimeticus*	Barradas et al. (2021)
54	Miranda et al.	2021	Korea	Asia	2015–2020	384	16	—	—	Miranda et al. (2021)
55	Mohammadian et al.	2021	Iran	Asia	216	201	20	Spring and Summer	—	Mohammadian, Noaman, and Emami (2021)
56	Calleja‐Bueno et al.	2022	Spain	Europe	2015	496	4	—	—	Calleja‐Bueno et al. (2022)
57	Altay et al.	2022	Kyrgyzstan	Asia	2017	358	6	—	—	Altay et al. (2022)
58	Zhang et al.	2022	China	Asia	2021–2022	940	55	Summer, Autumn and Winter	—	J. Zhang et al. (2022)
59	Poorghafoor Langroodi and Noaman	2022	Iran	Asia	2017	200	2	—	—	Langroodi and Noaman (2022)
60	Eleftheriou et al.	2022	USA	North America	2020–2021	327	2	Winter, Spring, Summer and Autumn	—	Eleftheriou et al. (2022)
61	Zobba et al.	2022	Senegal	Africa	2014	14	5	Summer	—	Zobba et al. (2022)
62	Noaman and Beiranvand	2022	Iran	Asia	—	200	4	—	—	V. Noaman and Beiranvand (2022)
63	Adjadj et al.	2023	Belgium	Europe	2019–2020	339	116	Winter	*Bois de Rotheux* *Bois d'Esneux* *Gratiebossen*	Adjadj et al. (2023)
64	Van Loo et al.	2023	Belgium	Europe	—	150	4	—	—	Van Loo et al. (2023)
65	Apaa et al.	2023	England	Europe	—	21	8	—	*Ixodes ricinus*	Apaa et al. (2023)
66	Mohanta et al.	2023	Bangladesh	Asia	2021–2022	276	2	Summer	—	Mohanta et al. (2023)
67	Persson Waller et al.	2023	Sweden	Europe	2020	30	11	Spring and Summer	—	P. Waller et al. (2023)
68	Zhou et al.	2023	China	Asia	2018–2020	176	7	Summer	—	S. Zhou et al. (2023)
69	Mitrea et al.	2024	Romania	Europe	—	21	2	—	—	Mitrea et al. (2024)
70	Ku et al.	2024	Korea	Asia	—	82	33	—	—	Ku et al. (2024)
71	Altay et al.	2024	Kyrgyzstan	Asia	—	531	17	—	—	Altay et al. (2024)
72	Chikufenji et al.	2024	Malawi	Africa	—	220	49	—	—	Chikufenji et al. (2024)

**TABLE 2:** Sub‐group analysis based on annual precipitation, humidity, annual rainfall, average temperature, climate, WHO regions, countries, continent, income level, Human Development Index (HDI), diagnostic method and seasons in included studies.

Variables	No studies	Sample size	Infected	Pooled prevalence (95% CI)	Heterogeneity		
					*I* ^2^	*τ* ^2^	*p* value
**Annual precipitation**							
<300	37	13,323	1286	8.2% (5%–12.1%)	97	3.3%	<0.001
300–650	21	5589	348	8.3% (3.2%–15.2%)	96	4.6%	<0.001
651–1000	10	2052	164	11.3% (1.9%–26.6%)	97	7.2%	<0.001
>1000	4	1503	133	6% (0%–28.1%)	98	3.6%	<0.001
Total	72	22,467	1931	8.5% (5.9%–11.5%)	97	4.1%	<0.001
**Humidity (%)**							
<40	9	3266	406	7% (2%–14.5%)	96	3.3%	<0.001
40–75	44	15,832	1217	8.1% (5%–11.8%)	97	3.8%	<0.001
>75	19	3369	308	10.3% (4.4%–17.9%)	96	5.3%	<0.001
Total	72	22,467	1931	8.5% (5.9%–11.5%)	97	4.1%	<0.001
**Annual rainfall (mm)**							
<400	16	4580	671	9.4% (4%–16.8%)	97	4.5%	<0.001
401–1000	37	11,992	930	9.5% (6%–13.8%)	97	3.6%	<0.001
1001–1500	16	5128	177	3.9% (1%–8.1%)	89	2.9%	<0.001
>1500	3	767	153	20.6% (0.3%–58.7%)	98	12.3%	<0.001
Total	72	22,467	1931	8.5% (5.9%–11.5%)	97	4.1%	<0.001
**Average temperature (°C)**							
<10	6	918	235	19.7% (5.4%–39.7%)	98	7%	<0.001
10–20	53	18,700	1461	7.5% (4.8%–10.7%)	97	3.7%	<0.001
>20	13	2995	235	8.1% (2.9%–15.2%)	95	3.7%	<0.001
Total	72	22,467	1931	8.5% (5.9%–11.5%)	97	4.1%	<0.001
**Climate**							
Oceanic climate	7	2003	156	9.9% (1.5%–23%)	98	4.7%	<0.001
Hot‐summer Mediterranean climate	10	3604	229	13.7% (4.7%–26.2%)	97	6.1%	<0.001
Tropical savanna climate	3	890	110	6.8% (0–25.6%)	98	5.3%	<0.001
Cold semi‐arid climates	10	3630	592	10% (2.8%–20.8%)	98	5.6%	<0.001
Humid subtropical climate	7	2155	75	4.5% (0.3%–11.9%)	70	2.7%	<0.001
Hot humid continental climate	28	8860	680	7.5% (4%–12%)	96	3.6%	<0.001
Hot desert climates	3	640	71	10.6% (6.5%–15.4%)	54	0.2%	<0.001
Hot semi‐arid climates	2	112	7	12.9% (0–57.1%)	91	10.3%	<0.001
Tropical rainforest climate	1	375	10	2.6% (0.1%–3%)	NA	NA	NA
Tropical monsoon climate	1	198	1	0.5% (0–2.7%)	NA	NA	NA
Total	72	22,467	1931	8.5% (5.9%–11.5%)	97	4.1%	<0.001
**WHO regions**							
African region	10	1884	135	11.3% (3.9%–21.5%)	94	4.3%	<0.001
Eastern Mediterranean region	13	4464	492	6.9% (3.2%–12%)	96	2.3%	<0.001
European region	23	7810	697	10.3% (5.6%–16.1%)	97	3.7%	<0.001
Region of the Americas	3	621	52	9.7% (0–49.7%)	98	16.4%	<0.001
South West Asian region	2	671	104	9.4% (0–45.7%)	99	9.4%	<0.001
Western Pacific region	19	6128	433	7.2% (2.7%–13.5%)	97	4.7%	<0.001
North and Central Asia	2	889	18	1.4% (0–5.4%)	91	0.6%	<0.001
Total	72	22,467	1931	8.5% (5.9%–11.5%)	97	4.1%	<0.001
**Countries**							
China	8	2678	124	5.8% (1.7%–11.7%)	88	0.2%	<0.001
Italy	4	1933	91	12.4% (2.8%–27%)	98	3.3%	<0.001
Turkey	4	2641	396	12.4% (2.8%–27.5%)	98	3.7%	<0.001
Spain	2	952	18	1.7% (0.2%–4.4%)	85	0.2%	0.010
Japan	4	1394	50	6.4% (0–24%)	81	5.6%	<0.001
Korea	6	1692	73	4.8% (0.3%–15.5%)	94	4.8%	<0.001
Iran	9	3266	406	7% (2%–14.5%)	96	3.3%	<0.001
Pakistan	2	598	21	3.9% (1.2%–7.7%)	71	0.2%	0.06
Iraq	2	600	65	10.1% (5.9%–15.2%)	71	0.2%	0.06
Kyrgyzstan	2	889	18	1.4% (0–5.4%)	91	0.6%	<0.001
Belgium	3	519	122	12.2% (0.3%–35%)	97	5.3%	<0.001
England	2	29	9	27.9% (7.1%–54.3%)	37	1.4%	<0.001
Bangladesh	2	671	104	9.4% (0–45.7%)	99	9.4%	<0.001
Sweden	2	101	21	23.5% (6%–47.3%)	83	2.5%	0.01
Ethiopia	1	457	19	4.1% (0%–2.3%)	NA	NA	NA
Serbia	1	163	4	2.4% (1%–6.2%)	NA	NA	NA
Germany	1	58	16	27.5% (16.7%–39.9%)	NA	NA	NA
Egypt	1	40	6	15% (8%–29.5%)	NA	NA	NA
Mozambique	1	219	6	2.7% (1%–5.6%)	NA	NA	NA
Puerto Rico	1	198	1	0.5% (0.1%–2.9%)	NA	NA	NA
Angola	1	98	2	2% (0.8%–7.4%)	NA	NA	NA
USA	1	327	2	0.6% (0.1%–2.1%)	NA	NA	NA
Senegal	1	14	5	35.7% (18.3%–60.5%)	NA	NA	NA
Romania	1	21	2	9.5% (4.4%–29.8%)	NA	NA	NA
Malawi	1	220	49	22.2% (17.3%–28.2%)	NA	NA	NA
Czech Republic	1	110	6	5.4% (2.8%–11.6%)	NA	NA	NA
Switzerland	1	285	2	0.7% (0%–0.9%)	NA	NA	NA
Norway	1	58	20	34.4% (23.9%–47.3%)	NA	NA	NA
Guatemala	1	96	49	51% (41.2%–60.7%)	NA	NA	NA
Uganda	1	375	10	2.6% (1.4%–4.8%)	NA	NA	NA
Algeria	1	36	15	41.6% (27.5%–57.6%)	NA	NA	NA
France	1	998	6	0.6% (0–0.5%)	NA	NA	NA
Tunisia	1	367	7	1.9% (0.8%–3.8%)	NA	NA	NA
Mongolia	1	364	186	51.1% (45.9%–56.2%)	NA	NA	NA
Total	72	22,467	1931	8.5% (5.9%–11.5%)	97	4.1%	<0.001
**HDI**							
Very high level	36	11,479	839	8.3% (4.7%–12.7%)	97	4.1%	<0.001
Medium level	10	2952	259	7.8% (2%–16.9%)	97	4.8%	<0.001
Low level	5	1285	89	9.1% (1.1%–22.7%)	94	4%	<0.001
High level	21	6751	744	9% (4.4%–15%)	97	4.3%	<0.001
Total	72	22,467	1931	8.5% (5.9%–11.5%)	97	4.1%	<0.001
**Diagnostic method**							
Direct smear	5	1443	180	8.6% (1.7%–19%)	96	2.3%	<0.001
Immunological techniques	16	4380	615	14.2% (6.5%–24.3%)	98	6.4%	<0.001
Molecular techniques	57	16,644	1136	7.7% (5%–7.9%)	96	3.8%	<0.001
Total	78	22,467	1931	8.5% (5.9%–11.5%)	97	4.1%	<0.001
**Season**							
Summer	9	1661	98	9.8% (1.5%–23%)	95	6.8%	<0.001
Spring	3	88	4	3.8% (0–12.5%)	12	0.6%	<0.001
Autumn	2	144	22	12.4% (0–53.3%)	96	10.2%	<0.001
Winter	2	437	118	14.5% (0–56.2%)	98	10.5%	<0.001
Spring and summer	6	714	97	16% (4.2%–33.1%)	95	5.7%	<0.001
Spring and autumn	1	1440	183	12.7% (10.8%–14.3%)	NA	NA	NA
Winter and spring	1	375	10	2.6% (1.4%–4.8%)	NA	NA	NA
Summer and autumn	1	389	3	0.7% (0.1%–2.1%)	NA	NA	NA
Autumn and winter	1	88	26	29.5% (21%–39.7%)	NA	NA	NA
Winter, spring and summer	2	630	35	7.9% (0–26.9%)	96	3.3%	<0.001
Spring, summer and autumn	3	1864	20	1% (0.4%–1.8%)	0	0	0.50
Summer, autumn and winter	1	940	55	5.8% (4.5%–7.5%)	NA	NA	NA
Winter, spring, summer and autumn	8	4219	810	17.1% (7%–30.4%)	98	5%	<0.001
Total	40	12,989	1481	8.5% (5.9%–11.5%)	97	4.1%	<0.001

### 2.3 Inclusion and Exclusion Criteria

The following inclusion criteria were incorporated for the current study: (1) The prevalence of *A. phagocytophilum* in cattle is reported in all published observational research, including cross‐sectional, case‐control and cohort studies; (2) articles with both full‐text and abstract available in English; (3) peer‐reviewed original research articles; (4) articles providing data on the total sample size and the number of positive cases; (5) studies published up until 2024; (6) studies that included non‐original data, review articles, case reports, case series, letters, editorials or publications with unclear or undetermined results were excluded from the analysis of this study.

Furthermore, publications that addressed *A. phagocytophilum* infection in humans or non‐cattle animals were not included.

### 2.4 Quality Assessment

The Newcastle–Ottawa Scale was used to assess the quality of the included studies (Table S1) (Abdoli, Olfatifar, Badri, et al. 2024; Abdoli, Olfatifar, Eslahi, et al. 2024a). The scoring system consisted of three factors: selection (up to 5 stars), comparability (up to 2 stars) and result (up to 3 stars).

### 2.5 Data Synthesis and Statistical Analysis

The global pooled prevalence of *A. phagocytophilum* in cattle was estimated with a 95% confidence interval (CI). A meta‐regression test was performed to examine the impact of the publication year of studies on prevalence. The publication bias was evaluated by the Luis Furuya‐Kanamori (LFK) index, Egger's plot and the Doi plot (Barendregt and Doi 2016; Abdoli, Olfatifar, Eslahi et al. 2024b). An LFK index within the range of outside ±2, ±2 and ±1 is regarded as significantly/major asymmetrical, slightly/minor asymmetrical and asymmetrical symmetrical (absence of publication bias), respectively. A Freeman–Tukey double arcsine transformation for the random‐effects model was applied to calculate the overall prevalence. Cochrane's Q test and inconsistency index (*I^2^* statistics) were used to assess heterogeneity among included studies, considering *I* ^2^ values of 25, 50 and 75% as low, medium and high heterogeneity, respectively. A *p* value lower than 0.05 was interpreted as statistically significant. All statistical analyses conducted herein were based on the meta‐package of R (version 3.6.1) (R Core Team 2016).

Sensitivity analysis was taken into consideration in this study to assess whether each study affected the overall results. After eliminating each study, this approach examines the total impact to identify any variations with the final outcome (Figure S1).

### 2.6 Predictions Analysis

We applied spline regression to predict future disease prevalence by modelling annual data from a meta‐analysis and generating corresponding forecasts. This approach effectively uncovered underlying patterns and accommodated non‐linear trends that simpler models might fail to capture. By precisely calibrating the smoothness parameter (degrees of freedom) in the spline function, we achieved a balance between bias and variance, which enhanced the model's predictive performance. Cross‐validation was employed to fine‐tune the degrees of freedom, ensuring optimal performance by minimizing the mean squared error (MSE).

## 3 Results

### 3.1 Literature Search Selection and Data Extraction

**FIGURE 1:**
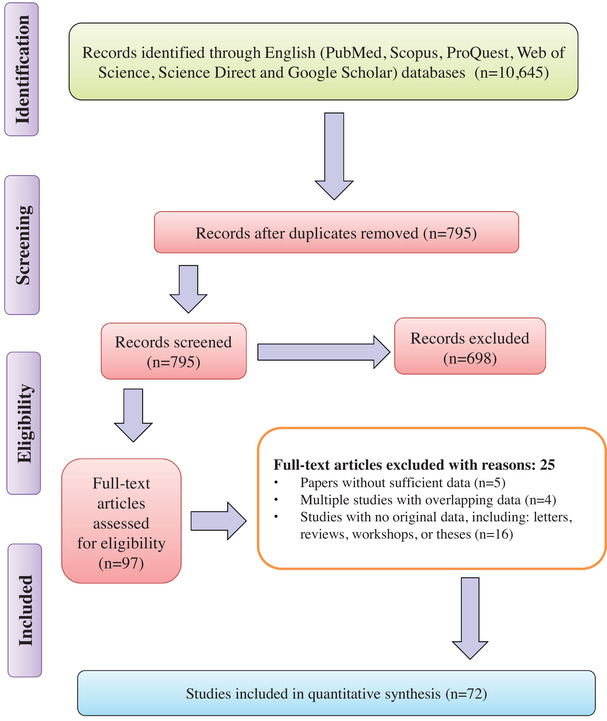
Flow diagram of the study design process.

A systematic search in this study identified 10,645 articles, of which 795 full‐text studies were assessed for eligibility. After evaluation, five studies were excluded due to insufficient data, four were removed for overlapping data, and 16 did not provide original data, including letters, reviews, workshops and theses. In the end, 72 studies met the eligibility criteria based on the critical appraisal standards (Figure 1).

### 3.2 Pooled Prevalence

**FIGURE 2:**
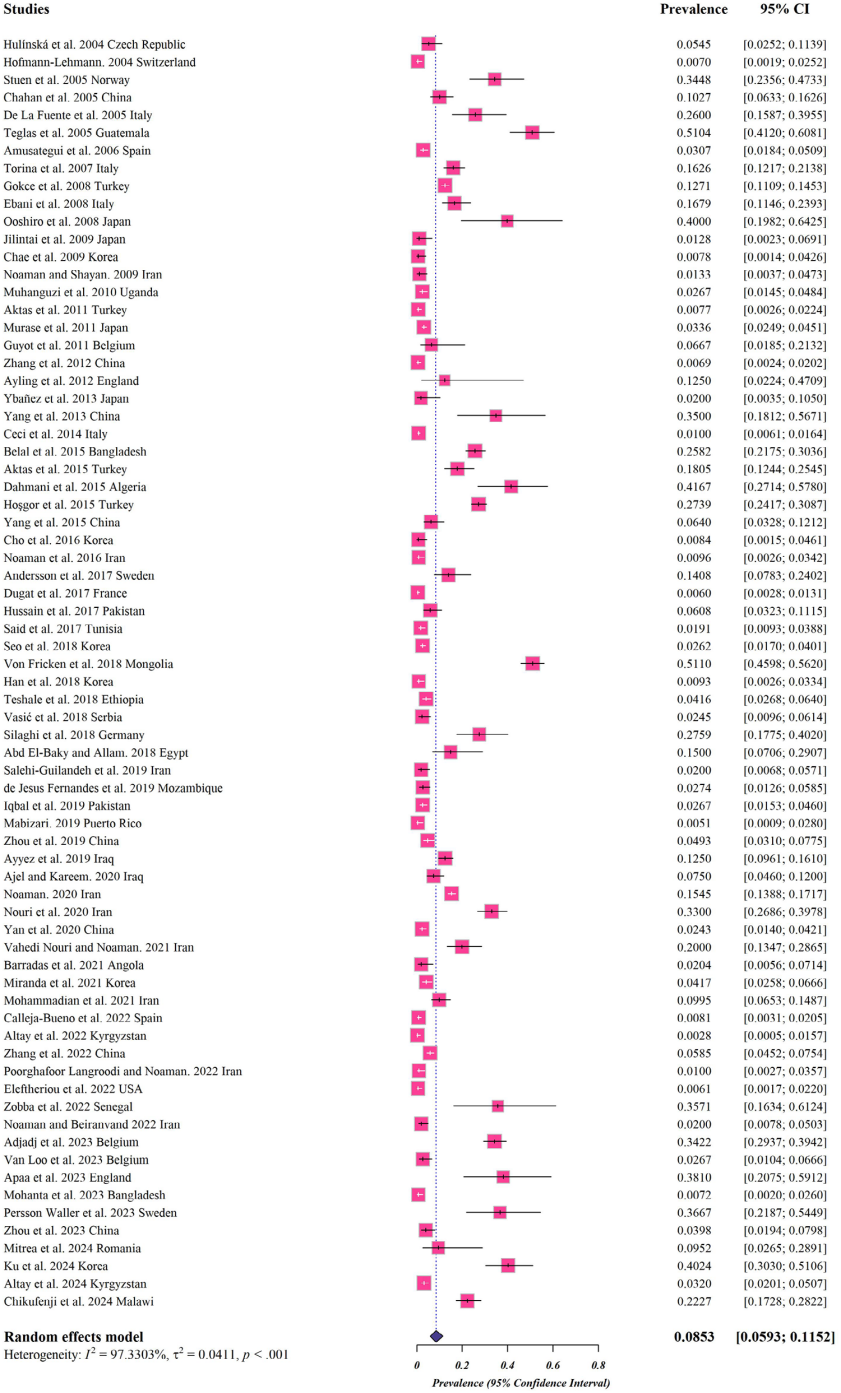
Forest plots for random‐effects meta‐analysis of *Anaplasma phagocytophilum* in cattle. (The boxes indicate the effect size of the studies (prevalence), and the whiskers indicate their confidence interval for corresponding effect size. There is no specific difference between white and black bars, only studies with a very narrow confidence interval are shown in white. In the case of diamond, their size indicates the size of the effect, and their length indicates confidence intervals.)

The pooled prevalence of *A. phagocytophilum* in cattle was (8.5%, 95% CI; 5.9%–11.5%) (Figure 2 and Table 2). The prevalence of *A. phagocytophilum* in cattle has been documented in 34 countries. Most publications were related to Iran (9 studies) followed by China (8 studies). The analysis based on the country illustrated that Mongolia (51.1%, 95% CI; 45.9%–56.2%), following Guatemala (51%, 95% CI; 41.2%–60.7%) both countries with one study had the highest pooled prevalence (Table 2).

**FIGURE 3:**
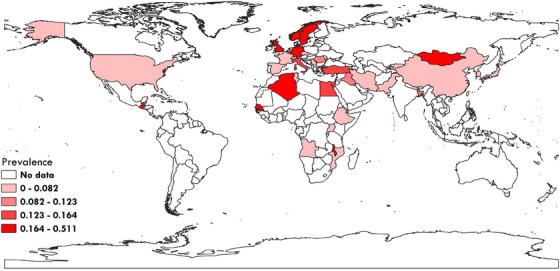
The global prevalence of *Anaplasma phagocytophilum* in cattle in different geographical regions of the world based on included studies.

According to the included studies, a map was created using QGIS3 software (https://qgis.org/en/site/ ) to demonstrate the prevalence of *A. phagocytophilum* in cattle in different geographical regions (Figure 3).

On the basis of WHO regions, African region showed the highest prevalence rate (11.3%, 95% CI; 3.9%–21.5%) (Table 2). According to the HDI, the highest pooled prevalence was related to the countries with the lowest HDI (9.1%, 95% CI; 1.1%–22.7%) (Table 2).

In terms of climate, our analysis indicated that the regions with a Hot‐summer Mediterranean climate exhibited the highest pooled prevalence (13.7%, 95% CI; 4.7%–26.2%), which had the highest pooled prevalence (Table 2).

Moreover, our analyses indicated that *A. phagocytophilum* in cattle was most prevalent in cattle in regions with annual rainfall of >1500 mm (20.62%, 95% CI; 0.3%–58.7%), annual precipitation of 651–1000 (11.3%, 95% CI; 1.9%–26.6%), humidity >75 (10.3%, 95% CI; 4.4%–17.9%) and average temperature <10°C (19.7%, 95% CI; 5.4–39.7) (Table 2).

Studies employing immunological techniques exhibited the highest pooled prevalence based on the detection method (14.2%, 95% CI; 6.5%–24.3%) (Table 2).

### 3.3 Publication Bias

**FIGURE 4:**
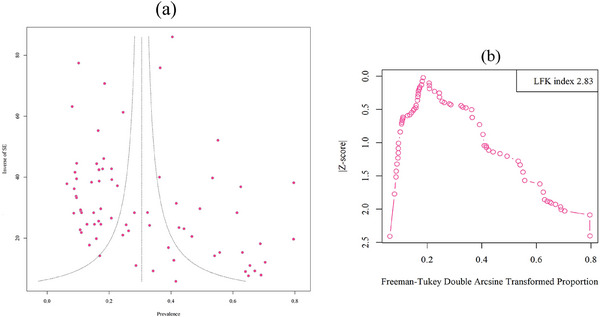
Egger's funnel plot to assess publication bias in studies evaluating of *Anaplasma phagocytophilum* in cattle (a). Doi plot: A Luis Furuya‐Kanamori (LFK) index 2.83 indicates major asymmetry (b).

Egger's test revealed the presence of a highly significant publication bias (*z* = 3.51, *p* = 0.0005). Furthermore, the Doi plot had a major asymmetry (LFK index: 2.83) (Figure 4).

### 3.4 Meta‐Regression

**FIGURE 5:**
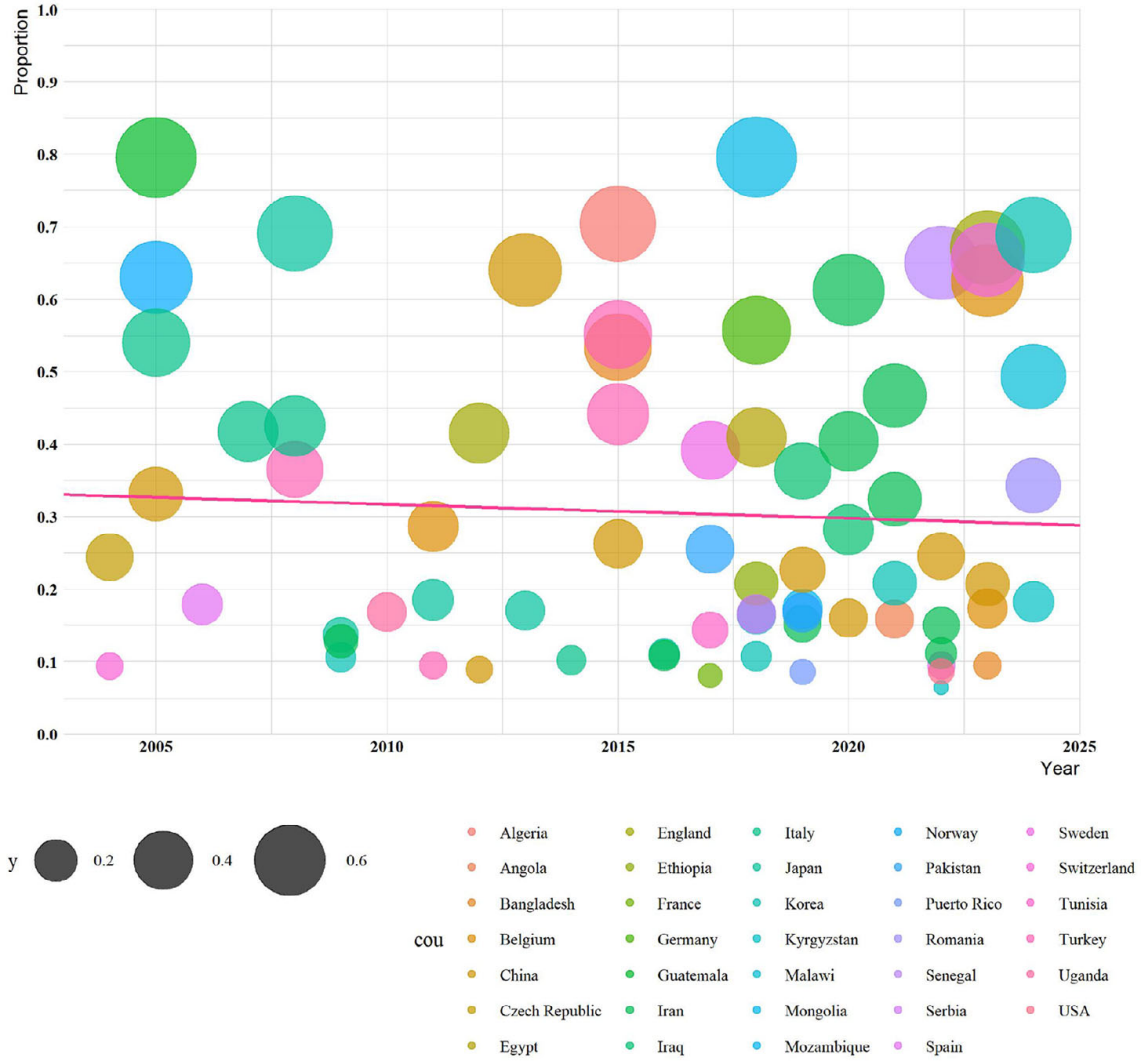
A meta‐regression graph for the prevalence of *Anaplasma phagocytophilum* in cattle based on year of publication. The pink line is the regression line, which was plotted on the basis of the intercept and the slope of the regression model. The different colour bubbles represent the countries under study, and their sizes indicate the effect size of each study.

The meta‐regression analysis demonstrated that there was no significant statistical relationship between the prevalence and the publication year (slope: 4.160, *p* = 0.623) (Figure 5).

### 3.5 Prediction Analysis

**FIGURE 6:**
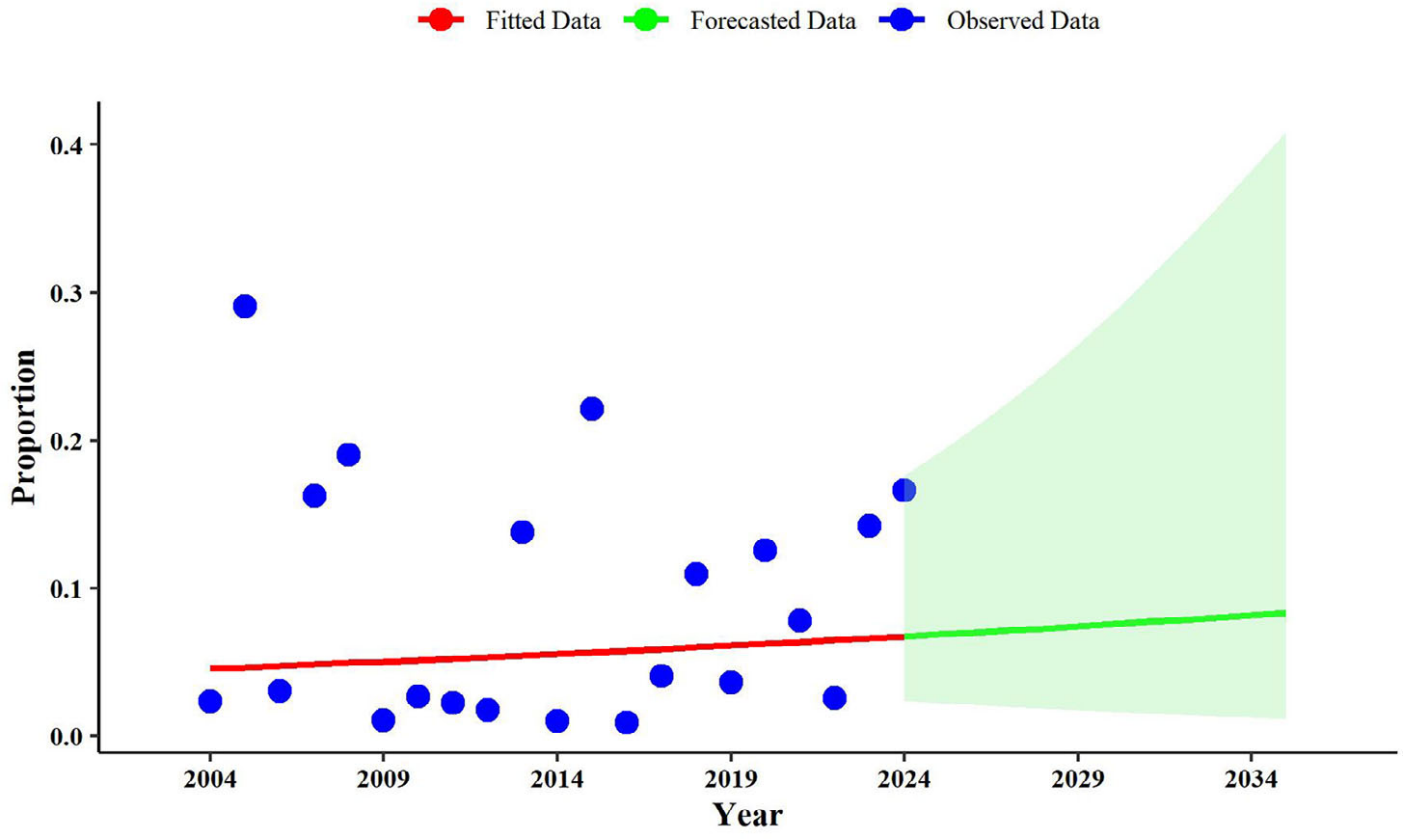
Prediction analysis of *Anaplasma phagocytophilum* in cattle up to 2035.

The annual predicted prevalence for 2024 was estimated at 0.067 (0.023–0.176), and this value is projected to increase by 2035 (0.083, 0.011–0.408) (Figure 6).

### 3.6 Quality Assessment

The quality assessment results revealed that out of the 72 studies evaluated, 26 scored between 4 and 6 points, indicating a moderate quality level, whereas the remaining 46 studies achieved scores ranging from 7 to 9 points, reflecting a high‐quality level (Table S1).

## 4 Discussion

Bovine anaplasmosis, caused by various *Anaplasma* species, including *A. phagocytophilum*, poses a considerable risk to cattle herds, affects the cattle industry's economic aspects and is prevalent in tropical and subtropical regions worldwide (Aktas and Çolak 2021). This study represents the first systematic review and meta‐analysis that focuses on the worldwide prevalence of *A. phagocytophilum* in cattle, incorporating data published during the first two decades of the 21st century.

The estimates based on the WHO region showed variations, with the highest prevalence in the African region (11.3%). This emphasizes the importance of understanding regional variations in prevalence for effective disease management and prevention. In Africa, where various tick‐borne diseases are widespread (Kocan, Blouin, and Barbet 2000), evidence for the presence of *A. phagocytophilum* remains scarce. This is likely due to the limited availability of advanced diagnostic tools. Conventional methods, such as microscopic analysis of stained blood smears and serological tests, lack the specificity to differentiate between species and require significant expertise and technical proficiency (Teshale et al. 2018). For these reasons, we believe that the actual prevalence might be even higher than currently reported.

Our country‐based analysis revealed that Mongolia, followed by Guatemala, displayed the highest prevalence of *A. phagocytophilum* in cattle compared to other countries.

Mongolia, located in eastern and central Asia, is a landlocked country with a population of 3.1 million. It shares its borders with Russia to the north and the People's Republic of China to the south, east and west (Narankhajid et al. 2018). Elevated seroprevalence of *A. phagocytophilum* in Mongolia is a serious issue, primarily due to the potential of the bacterium to induce severe HGA as well as complications such as spontaneous abortion, TBF and lethargy in livestock (von Fricken et al. 2018). In a prior serosurvey conducted in Khuvsgul and Khentii, the northern provinces of Mongolia, free‐ranging livestock were examined for *Anaplasma* and spotted fever group (SFG) *Rickettsia*. The results revealed that 35.8% tested positive for *Anaplasma* and 21.6% for SFG *Rickettsia* (Papageorgiou et al. 2012).

Furthermore, the presence of *A. phagocytophilum* in ticks and documented clinical cases of anaplasmosis in close proximity to the Mongolian border underscore the need for additional investigations in Mongolia (von Fricken et al. 2018).

Guatemala, situated in Central America, is an endemic region for tick‐borne diseases including *Babesia* and *Anaplasma* (Teglas et al. 2005). The Peten region in Guatemala experiences frequent and extensive tick infestations in both horses and cattle, presenting a significant risk for transmitting tick‐borne diseases to the local livestock and potentially the area's human population (Teglas et al. 2005). *Rhipicephalus* (*Boophilus*) *microplus* and *Amblyomma cajennense* are ticks species that are most frequently observed on cattle and are commonly linked to infestations in both cattle and horses in Central America, with *B. microplus* being notably prevalent on cattle based on numerous reports (Alvarez, Bonilla, and Chacón 2000; Payne and Scott 1982).

Guatemala, besides Honduras, is among countries with the lowest HDI scores and the highest proportion of poverty and child malnutrition in Central America, contributing the most annual cases of neglected tropical diseases (NTDs) documented in the region. Moreover, countries in Central America have a history of various infectious diseases notably many NTDs affecting a large number of people (Dye‐Braumuller et al. 2022).

Herein, we demonstrated that regions with a hot‐summer Mediterranean climate were accountable for the highest pooled prevalence and had the highest overall prevalence. Ticks and tick‐borne diseases (TTBDs) are highly susceptible to climate change because environmental conditions influence the stages of their life cycle. The warming climate has created favourable conditions for ticks to thrive and reproduce, resulting in faster development and an accelerated lifecycle. However, prolonged extreme temperature fluctuations, low humidity and heavy rainfall can negatively affect tick development by decreasing their activity and increasing their mortality rate (Ogden et al. 2021).

On the basis of our findings, immunological techniques were linked to the highest prevalence rates. Ab‐IFAT has been widely utilized in epidemiological investigations, and competitive ELISA (cELISA) offers excellent sensitivity and specificity for detecting *Anaplasma*‐specific antibodies (Athanasiou et al. 2022). However, these techniques are associated with the probability of cross‐reactivity between *A. phagocytophilum* and other species of bovine anaplasmosis (Aubry and Geale 2011). Furthermore, serology is not applicable shortly after the occurrence of infection due to the time that needs to be passed between infection and the development of antibodies in peripheral blood (Athanasiou et al. 2022).

There are a number of benefits to using PCR‐based diagnostic assays rather than conventional serologic and blood smear tests for the identification of these infections. When set up in multiplexed reactions, PCR tests can detect coinfections and have nearly 100% sensitivity and specificity rates with a higher sensitivity during the acute stage of infection as well (Schotthoefer et al. 2013).

A real‐time combined PCR is introduced via previous research, which represents an alternative technique to serologic and blood smear tests. A real‐time combined PCR, which represents an alternative technique to serologic and blood smear tests, is introduced via previous research. This method detects and differentiates at least four species in a single reaction assay. Hence, this method can be helpful for accurate diagnosis in regions where the distributions of these pathogens may overlap (Bell and Patel 2005).

## 5 Limitations

The limitations of our study include the potential impact of publication bias, which may have arisen due to the limited number of reports and the absence of data from certain geographic regions. Additionally, this study was restricted to English‐language publications. Many of the studies included in the analysis relied on direct blood smears as a diagnostic tool, which could lead to reduced sensitivity and specificity. Furthermore, a large number of studies did not identify the pathogen at the species level. Despite these limitations, our review and meta‐analysis offer the most comprehensive global estimates of *A. phagocytophilum* prevalence in cattle.

## 6 Conclusion

This study shed light on the worldwide prevalence of *A. phagocytophilum* in cattle, emphasizing its significance as a significant tick‐borne disease with substantial economic implications for the cattle industry. The pathogen's wide distribution and association with various tick species underscore the complexity of its epidemiology. Our study revealed variations in the pooled prevalence rate across different regions and climates, highlighting the need for region‐specific disease management strategies. Climate, specifically a hot‐summer Mediterranean climate, was identified as contributing to the prevalence of *A. phagocytophilum*.

Understanding the dynamics of *A. phagocytophilum* in cattle is crucial for implementing effective control and prevention measures to mitigate its effect on animal health and economic productivity in the agricultural sector. As the first systematic review and meta‐analysis focusing on the global prevalence of the infection in cattle, this research not only consolidates existing knowledge but also sets the stage for future advancements in veterinary research and public health.

## Author Contributions

**Amir Abdoli**: conceptualization, project administration, writing – review and editing, supervision. **Meysam Olfatifar**: methodology, data curation, formal analysis, software. **Leila Zaki**: investigation, visualization. **Farhad Nikkhahi**: visualization, supervision. **Fatemeh Fardsanei**: visualization, supervision. **Sona Sobhani**: investigation. **Hamid Sadeghi**: visualization. **Aida Vafae Eslahi**: conceptualization, investigation, writing – original draft, writing – review and editing. **Milad Badri**: conceptualization, project administration, methodology, data curation, visualization, writing – original draft, writing – review and editing, supervision. All the authors commented on the drafts of the manuscript and approved the final version of the article.

## Ethics Statement

The ethical approval was required and provided for this study, as stated by our institutional review board (no. IR.QUMS.REC.1402.365).

## Conflicts of Interest

The authors declare no conflicts of interest.

### Peer Review

The peer review history for this article is available at https://publons.com/publon/10.1002/vms3.70251.

## Supporting information



Supporting Information

Supporting Information

## Data Availability

The authors have nothing to report.
