# Supplementary material for: Global Prevalence of Anaplasma phagocytophilum in Cattle: A One Health Perspective, Meta‐Analysis and Future Predictions (up to 2035)
Source: Vet Med Sci. 2025 Feb 19;11(2):e70251. doi: 10.1002/vms3.70251 (PMC11837283; doi:10.1002/vms3.70251)
Supplement: Supplementary file 1 — Supporting Information [file VMS3-11-e70251-s001.pdf]

Studies	Prevalence	95% CI
Omitting Hulínská et al. 2004 Czech Republic	0.0858	[0.0594; 0.1162]
Omitting Hofmann-Lehmann. 2004 Switzerland	0.0870	[0.0606; 0.1174]
Omitting Stuen et al. 2005 Norway	0.0827	[0.0572; 0.1121]
Omitting Chahan et al. 2005 China	0.0851	[0.0588; 0.1154]
Omitting De La Fuente et al. 2005 Italy	0.0835	[0.0577; 0.1133]
Omitting Teglas et al. 2005 Guatemala	0.0810	[0.0563; 0.1095]
Omitting Amusategui et al. 2006 Spain	0.0863	[0.0599; 0.1167]
Omitting Torina et al. 2007 Italy	0.0843	[0.0582; 0.1145]
Omitting Gokce et al. 2008 Turkey	0.0847	[0.0585; 0.1150]
Omitting Ebani et al. 2008 Italy	0.0843	[0.0582; 0.1144]
Omitting Ooshiro et al. 2008 Japan	0.0831	[0.0577; 0.1124]
Omitting Jilintai et al. 2009 Japan	0.0867	[0.0602; 0.1170]
Omitting Chae et al. 2009 Korea	0.0869	[0.0605; 0.1172]
Omitting Noaman and Shayan. 2009 Iran	0.0867	[0.0603; 0.1171]
Omitting Muhanguzi et al. 2010 Uganda	0.0864	[0.0600; 0.1168]
Omitting Aktas et al. 2011 Turkey	0.0870	[0.0606; 0.1174]
Omitting Murase et al. 2011 Japan	0.0863	[0.0598; 0.1167]
Omitting Guyot et al. 2011 Belgium	0.0856	[0.0592; 0.1159]
Omitting Zhang et al. 2012 China	0.0871	[0.0607; 0.1174]
Omitting Ayling et al. 2012 England	0.0854	[0.0593; 0.1155]
Omitting Ybañez et al. 2013 Japan	0.0864	[0.0600; 0.1168]
Omitting Yang et al. 2013 China	0.0832	[0.0576; 0.1127]
Omitting Ceci et al. 2014 Italy	0.0870	[0.0606; 0.1173]
Omitting Belal et al. 2015 Bangladesh	0.0833	[0.0574; 0.1130]
Omitting Aktas et al. 2015 Turkey	0.0842	[0.0580; 0.1142]
Omitting Dahmani et al. 2015 Algeria	0.0823	[0.0570; 0.1114]
Omitting Hoşgor et al. 2015 Turkey	0.0831	[0.0573; 0.1128]
Omitting Yang et al. 2015 China	0.0857	[0.0593; 0.1160]
Omitting Cho et al. 2016 Korea	0.0869	[0.0605; 0.1172]
Omitting Noaman et al. 2016 Iran	0.0869	[0.0605; 0.1172]
Omitting Andersson et al. 2017 Sweden	0.0846	[0.0584; 0.1148]
Omitting Dugat et al. 2017 France	0.0871	[0.0607; 0.1175]
Omitting Hussain et al. 2017 Pakistan	0.0857	[0.0593; 0.1161]
Omitting Said et al. 2017 Tunisia	0.0866	[0.0602; 0.1170]
Omitting Seo et al. 2018 Korea	0.0865	[0.0600; 0.1169]
Omitting Von Fricken et al. 2018 Mongolia	0.0808	[0.0561; 0.1091]
Omitting Han et al. 2018 Korea	0.0869	[0.0605; 0.1173]
Omitting Teshale et al. 2018 Ethiopia	0.0861	[0.0597; 0.1165]
Omitting Vasić et al. 2018 Serbia	0.0864	[0.0600; 0.1168]
Omitting Silaghi et al. 2018 Germany	0.0833	[0.0575; 0.1130]
Omitting Abd El-Baky and Allam. 2018 Egypt	0.0846	[0.0584; 0.1148]
Omitting Salehi-Guilandeh et al. 2019 Iran	0.0865	[0.0601; 0.1169]
Omitting de Jesus Fernandes et al. 2019 Mozambique	0.0864	[0.0600; 0.1168]
Omitting Iqbal et al. 2019 Pakistan	0.0864	[0.0600; 0.1168]
Omitting Mabizari. 2019 Puerto Rico	0.0871	[0.0607; 0.1174]
Omitting Zhou et al. 2019 China	0.0859	[0.0595; 0.1163]
Omitting Ayyez et al. 2019 Iraq	0.0848	[0.0585; 0.1150]
Omitting Ajel and Kareem. 2020 Iraq	0.0855	[0.0591; 0.1159]
Omitting Noaman. 2020 Iran	0.0844	[0.0582; 0.1146]
Omitting Nouri et al. 2020 Iran	0.0826	[0.0570; 0.1120]
Omitting Yan et al. 2020 China	0.0865	[0.0601; 0.1169]
Omitting Vahedi Nouri and Noaman. 2021 Iran	0.0840	[0.0579; 0.1140]
Omitting Barradas et al. 2021 Angola	0.0865	[0.0601; 0.1169]
Omitting Miranda et al. 2021 Korea	0.0861	[0.0597; 0.1165]
Omitting Mohammadian et al. 2021 Iran	0.0851	[0.0588; 0.1155]
Omitting Calleja-Bueno et al. 2022 Spain	0.0870	[0.0606; 0.1174]
Omitting Altay et al. 2022 Kyrgyzstan	0.0873	[0.0609; 0.1176]
Omitting Zhang et al. 2022 China	0.0858	[0.0594; 0.1162]
Omitting Poorghafoor Langroodi and Noaman. 2022 Iran	0.0869	[0.0605; 0.1172]
Omitting Eleftheriou et al. 2022 USA	0.0871	[0.0607; 0.1174]
Omitting Zobba et al. 2022 Senegal	0.0835	[0.0579; 0.1129]
Omitting Noaman and Beiranvand 2022 Iran	0.0866	[0.0601; 0.1170]
Omitting Adjadj et al. 2023 Belgium	0.0824	[0.0569; 0.1118]
Omitting Van Loo et al. 2023 Belgium	0.0864	[0.0600; 0.1168]
Omitting Apaa et al. 2023 England	0.0829	[0.0575; 0.1123]
Omitting Mohanta et al. 2023 Bangladesh	0.0870	[0.0606; 0.1174]
Omitting Persson Waller et al. 2023 Sweden	0.0828	[0.0573; 0.1122]
Omitting Zhou et al. 2023 China	0.0861	[0.0597; 0.1165]
Omitting Mitrea et al. 2024 Romania	0.0853	[0.0590; 0.1155]
Omitting Ku et al. 2024 Korea	0.0821	[0.0568; 0.1112]
Omitting Altay et al. 2024 Kyrgyzstan	0.0863	[0.0599; 0.1167]
Omitting Chikufenji et al. 2024 Malawi	0.0837	[0.0577; 0.1136]
**Random effects model**	**0.0853**	**[0.0593; 0.1152]**

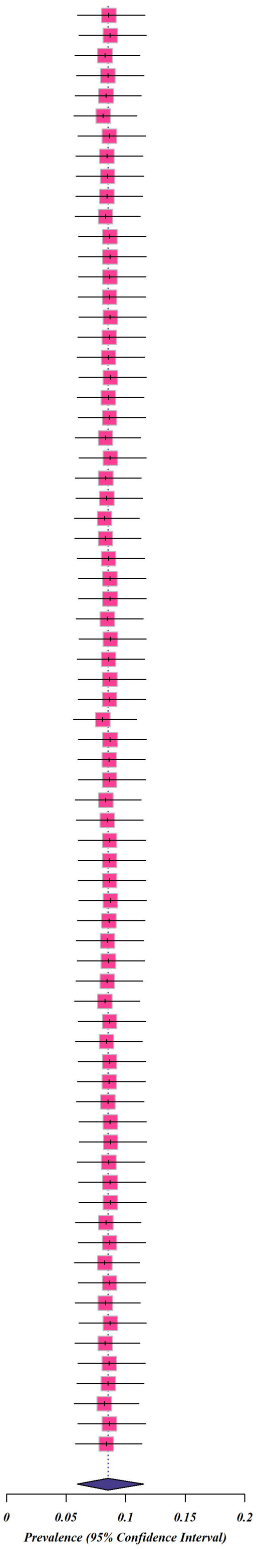

Supplementary Figure 1. Sensitivity analysis for assessing the effect of each primary study on the total estimates.
