# Supplementary material for: Global Prevalence of Anaplasma phagocytophilum in Cattle: A One Health Perspective, Meta‐Analysis and Future Predictions (up to 2035)
Source: Vet Med Sci. 2025 Feb 19;11(2):e70251. doi: 10.1002/vms3.70251 (PMC11837283; doi:10.1002/vms3.70251)
Supplement: Supplementary file 2 — Supporting Information [file VMS3-11-e70251-s002.docx]

**Supplementary Table 1.** Quality assessment using the Newcastle–Ottawa scale modified for cross sectional studies.

| Study No. | Author name | Publication years | Country | Selection  (maximum of 5 stars) | Comparability  (maximum of 2 stars) | Outcome  (maximum of 3 stars) | Total Score |
| --- | --- | --- | --- | --- | --- | --- | --- |
| 1 | Hulínská et al. | 2004 | Czech Republic | *** | * | *** | 7 |
| 2 | Hofmann-Lehmann, | 2004 | Switzerland | ** | * | ** | 5 |
| 3 | Stuen et al. | 2005 | Norway | *** | * | ** | 6 |
| 4 | Chahan et al. | 2005 | China | *** | * | *** | 7 |
| 5 | De La Fuente et al. | 2005 | Italy | *** | * | *** | 7 |
| 6 | Teglas et al. | 2005 | Guatemala | **** | ** | ** | 8 |
| 7 | Amusategui et al. | 2006 | Spain | *** | * | ** | 6 |
| 8 | Torina et al. | 2007 | Italy | ** | * | ** | 5 |
| 9 | Gokce et al. | 2008 | Turkey | *** | * | *** | 7 |
| 10 | Ebani et al. | 2008 | Italy | ** | * | ** | 5 |
| 11 | Ooshiro et al. | 2008 | Japan | *** | ** | *** | 8 |
| 12 | Jilintai et al. | 2009 | Japan | *** | * | *** | 7 |
| 13 | Chae et al. | 2009 | Korea | *** | ** | *** | 8 |
| 14 | Noaman and Shayan. | 2009 | Iran | *** | * | ** | 6 |
| 15 | Muhanguzi et al. | 2010 | Uganda | **** | ** | ** | 8 |
| 16 | Aktas et al. | 2011 | Turkey | *** | * | ** | 6 |
| 17 | Murase et al. | 2011 | Japan | ** | * | ** | 5 |
| 18 | Guyot et al. | 2011 | Belgium | *** | * | *** | 7 |
| 19 | Zhang et al. | 2012 | China | *** | ** | *** | 8 |
| 20 | Ayling et al. | 2012 | England | ** | * | ** | 5 |
| 21 | Ybañez et al. | 2013 | Japan | *** | * | *** | 7 |
| 22 | Yang et al. | 2013 | China | *** | ** | *** | 8 |
| 23 | Ceci et al. | 2014 | Italy | *** | ** | *** | 8 |
| 24 | Belal et al. | 2015 | Bangladesh | ** | ** | *** | 7 |
| 25 | Aktas et al. | 2015 | Turkey | *** | ** | ** | 7 |
| 26 | Dahmani et al. | 2015 | Algeria | ** | ** | ** | 6 |
| 27 | Hoşgor et al. | 2015 | Turkey | *** | * | *** | 7 |
| 28 | Yang et al. | 2015 | China | *** | ** | *** | 8 |
| 29 | Cho et al. | 2016 | Korea | ** | * | ** | 5 |
| 30 | Noaman et al. | 2016 | Iran | *** | ** | *** | 8 |
| 31 | Andersson et al. | 2017 | Sweden | ** | ** | ** | 6 |
| 32 | Dugat et al. | 2017 | France | ** | ** | *** | 7 |
| 33 | Hussain et al. | 2017 | Pakistan | ** | ** | *** | 7 |
| 34 | Said et al. | 2017 | Tunisia | ** | ** | ** | 6 |
| 35 | Seo et al. | 2018 | Korea | **** | * | *** | 8 |
| 36 | Von Fricken et al. | 2018 | Mongolia | **** | ** | *** | 9 |
| 37 | Han et al. | 2018 | Korea | *** | ** | *** | 8 |
| 38 | Teshale et al. | 2018 | Ethiopia | ** | ** | ** | 6 |
| 39 | Vasić et al. | 2018 | Serbia | ** | * | ** | 5 |
| 40 | Silaghi et al. | 2018 | Germany | *** | ** | *** | 8 |
| 41 | Abd El-Baky and Allam. | 2018 | Egypt | ** | ** | ** | 6 |
| 42 | Salehi-Guilandeh et al. | 2019 | Iran | *** | * | ** | 6 |
| 43 | de Jesus Fernandes et al. | 2019 | Mozambique | **** | ** | *** | 9 |
| 44 | Iqbal et al. | 2019 | Pakistan | ** | ** | ** | 6 |
| 45 | Mabizari. | 2019 | Puerto Rico | **** | ** | *** | 9 |
| 46 | Zhou et al. | 2019 | China | ** | ** | *** | 7 |
| 47 | Ayyez et al. | 2019 | Iraq | **** | * | ** | 7 |
| 48 | Yan et al. | 2020 | China | ** | ** | ** | 6 |
| 49 | Ajel and Kareem. | 2020 | Iraq | **** | ** | *** | 9 |
| 50 | Noaman. | 2020 | Iran | *** | * | *** | 7 |
| 51 | Nouri et al. | 2020 | Iran | **** | ** | *** | 9 |
| 52 | Vahedi Nouri and Noaman. | 2021 | Iran | ** | ** | *** | 7 |
| 53 | Barradas et al. | 2021 | Angola | *** | ** | *** | 8 |
| 54 | Miranda et al. | 2021 | Korea | *** | * | *** | 7 |
| 55 | Mohammadian et al. | 2021 | Iran | ** | ** | *** | 7 |
| 56 | Calleja‐Bueno etal. | 2022 | Spain | ** | ** | ** | 6 |
| 57 | Altay et al. | 2022 | Kyrgyzstan | ** | * | ** | 5 |
| 58 | Zhang et al. | 2022 | China | **** | ** | ** | 8 |
| 59 | Poorghafoor Langroodi and Noaman. | 2022 | Iran | **** | ** | *** | 9 |
| 60 | Eleftheriou et al. | 2022 | USA | ** | * | ** | 5 |
| 61 | Zobba et al. | 2022 | Senegal | *** | ** | *** | 8 |
| 62 | Noaman & Beiranvand | 2022 | Iran | ** | ** | ** | 6 |
| 63 | Adjadj et al. | 2023 | Belgium | ** | ** | *** | 7 |
| 64 | Van Loo et al. | 2023 | Belgium | **** | ** | *** | 9 |
| 65 | Apaa et al. | 2023 | England | ** | ** | *** | 7 |
| 66 | Mohanta et al. | 2023 | Bangladesh | ** | ** | ** | 6 |
| 67 | Persson Waller et al. | 2023 | Sweden | ** | ** | *** | 7 |
| 68 | Zhou et al. | 2023 | China | *** | ** | *** | 8 |
| 69 | Mitrea et al. | 2024 | Romania | ** | ** | ** | 6 |
| 70 | Ku et al. | 2024 | Korea | *** | * | ** | 6 |
| 71 | Altay et al. | 2024 | Kyrgyzstan | **** | ** | *** | 9 |
| 72 | Chikufenji et al. | 2024 | Malawi | ** | ** | *** | 7 |

*Indicates one criteria was followed, ** two criteria were followed, ***three criteria were followed, ****four criteria were followed, and ***** five criteria were followed.
